# New Sanatoria

**Published:** 1921-01-29

**Authors:** 


### New Sanatoria.

After considerable discussion the Nottingham City Council has adopted the proposal of the Health Committee to establish a sanatorium on the Bulwell Hall estate, at an estimated cost of £100,000. The estate, which is the property of the Council, is now under cultivation, and thirty-four acres will be devoted to the sanatorium.

The alterations at the sanatorium known as the Harts, Woodford, acquired by the Borough of East Ham, have cost about £20,000, and the house, which stands in thirty acres, £10,000. A pavilion, with twenty-four beds for incipient cases, will be built later. The grounds are not only sheltered but beautiful, and include meadows, walled gardens, and woodland, with many walks, including a shady path, a mile long, round the estate. Advanced cases will also be received, and the conservatory will be reserved for patients in cold weather. On the opening day between twenty and thirty patients were under treatment. A doctor's house is to be built on the property.

